# A Soft Robotic Wearable Wrist Device for Kinesthetic Haptic Feedback

**DOI:** 10.3389/frobt.2018.00083

**Published:** 2018-07-24

**Authors:** Erik H. Skorina, Ming Luo, Cagdas D. Onal

**Affiliations:** Soft Robotics Lab, Worcester Polytechnic Institute, Worcester, MA, United States

**Keywords:** soft robotics, wearable devices, haptics, pneumatics, human-robot interaction

## Abstract

Advances in soft robotics provide a unique approach for delivering haptic feedback to a user by a soft wearable device. Such devices can apply forces directly on the human joints, while still maintaining the safety and flexibility necessary for use in close proximity to the human body. To take advantage of these properties, we present a new haptic wrist device using pressure-driven soft actuators called reverse pneumatic artificial muscles (rPAMs) mounted on four sides of the wrist. These actuators are originally pre-strained and release compressive stress under pressure, applying a safe torque around the wrist joints while being compact and portable, representing the first soft haptic device capable of real-time feedback. To demonstrate the functional utility of this device, we created a virtual path-following task, wherein the user employs the motion of their wrist to control their embodied agent. We used the haptic wrist device to assist the user in following the path and study their performance with and without haptic feedback in multiple scenarios. Our results quantify the effect of wearable soft robotic haptic feedback on user performance. Specifically, we observed that our haptic feedback system improved the performance of users following complicated paths in a statistically significant manner, but did not show improvement for simple linear paths. Based on our findings, we anticipate broader applications of wearable soft robotic haptic devices toward intuitive user interactions with robots, computers, and other users.

## 1. Introduction

As computers become increasingly prevalent, the ability of humans and computers to communicate becomes more important. While much can be conveyed visually, humans have access to other senses that can be used to communicate information and provide feedback to a human user.

Haptic feedback devices have been used to convey subtle informational cues to users. These come in two basic categories: tactile and kinesthetic. Tactile haptic feedback uses purely sensory cues, such as vibrations, to inform the user of events or provide the illusion of forces, as in Amemiya and Gomi (2014). This has been used for a range of activities, such as gait training (Dowling et al., 2010) and vision aid (Johnson and Higgins, 2006).

Kinesthetic haptic feedback, which this paper focuses on, utilizes real forces in order provide feedback to the user. The simplest example of this is the force-feedback-enabled joystick. Chciuk et al. (2017) used a force-feedback joystick to teleoperate a robotic arm, and showed that the physical feedback provided a significant improvement to the performance of the user. Another example of a force-feedback joystick was developed by Riecke et al. (2016) for teleoporating robots in space. This system was developed to use the rapid and intuitive flow of information from the force feedback joystick to the operator to compensate for the communication lag in long-distance robot teleoperation. These joystick systems can provide strong, high-bandwidth feedback, but require the user to manipulate an external device. Other applications of rigid, kinesthetic feedback include robotic surgery as discussed by Wagner et al. (2002), where haptic feedback reduced errors by a factor of 3. In addition, work has been done by Metzger et al. (2012) using kinesthetic feedback for rehabilitation.

Similar electric motor techniques can be directly applied to the user's joints. Margineanu et al. (2018) created a 5-DoF haptic arm exoskeleton for use in space telerobotics. This system was effective, but was heavy and bulky. MA and Ben-Tzvi (2015) developed an exoskeletal glove which used rigid links protruding above the hand to apply forces to the user's fingertips. This method is bulky, and can easily become a hindrance when not used in a lab setting. Blake and Gurocak (2009) developed a similar glove, but used magnetorheological fluids to convey variable stiffness information to the user. Bouzit et al. (2002) developed similar device, but one that used pneumatic pistons to apply the forces. An example of a haptic wrist actuator was presented by Erwin et al. (2016). This device used piezoelectric actuators to provide relatively compliant (and Magnetic Resonance Imaging compatible) forces, but required a complicated mechanism, making it suited for use in controlled environments. A hybrid technique combining properties of tactile and kinesthetic was used by Schorr et al. (2013). This work uses a motor to physically stretch the skin of the user to simulate force effects, representing a skin-based haptic feedback that nevertheless involves real forces.

These haptic technologies rely on the user manipulating an external device or being constrained by an inflexible joint. However, there is a significant discrepancy in this approach, since human bodies are soft and flexible. Thus, in order to provide a seamless experience for haptic interactions, we posit that it would be appropriate for haptic devices to be soft and flexible to match the mechanical behavior of the human body. Soft feedback can be applied to the user in many ways, including using soft pneumatic actuators. One example of a soft pneumatic actuator is the McKibben muscle (or the pneumatic artificial muscle) as discussed by Chou and Hannaford (1996). This actuator type consists of a rubber tube wrapped in a mesh which causes it to contract when pressurized. McKibben muscles were used by Jadhav et al. (2015) as part of a soft glove for haptic feedback for a piano-playing VR experience. The soft actuators provided safe forces to the user's fingers, while the compliant nature of the glove made it comfortable and adaptable for a wide range of hand sizes. Patterson and Katz (1992) compared several haptic feedback devices used in active-prosthetic grasping. They found that pneumatic feedback was more reliable and easy to interpret than vibro-tactile haptic or visual feedback. Soft techniques can also be used for purely tactile feedback. One example is the work of Koo et al. (2008), where the authors used a series of small electroactive polymer nodules to apply stimulation to the user without additional electromechanical transmissions.

In this paper, we debut a novel wearable soft haptic wrist device capable of applying feedback to the user in the form of real torques around the wrist. We use soft linear actuators we call reverse pneumatic artificial muscles (rPAMs). These actuators consist of tubes of silicone rubber wrapped in thin helical thread such that when pressurized, they extend. This extension makes them more efficient than McKibben Muscles, which contract axially and expand radially when pressured. We investigated the performance of these actuators operating antagonistically in Skorina et al. (2015), with an actuator mounted on either side of a revolute joint analogous to a wrist. We also performed similar experiments using rPAM actuators as part of a bidirectional bending segment in Luo et al. (2017). In both of these works, we used valve PWM (Pulse-Width Modulation) to approximate pressure control, a technique idea for wearable devices, and could perform precise position control at up to 6 Hz.

**Figure 1:**
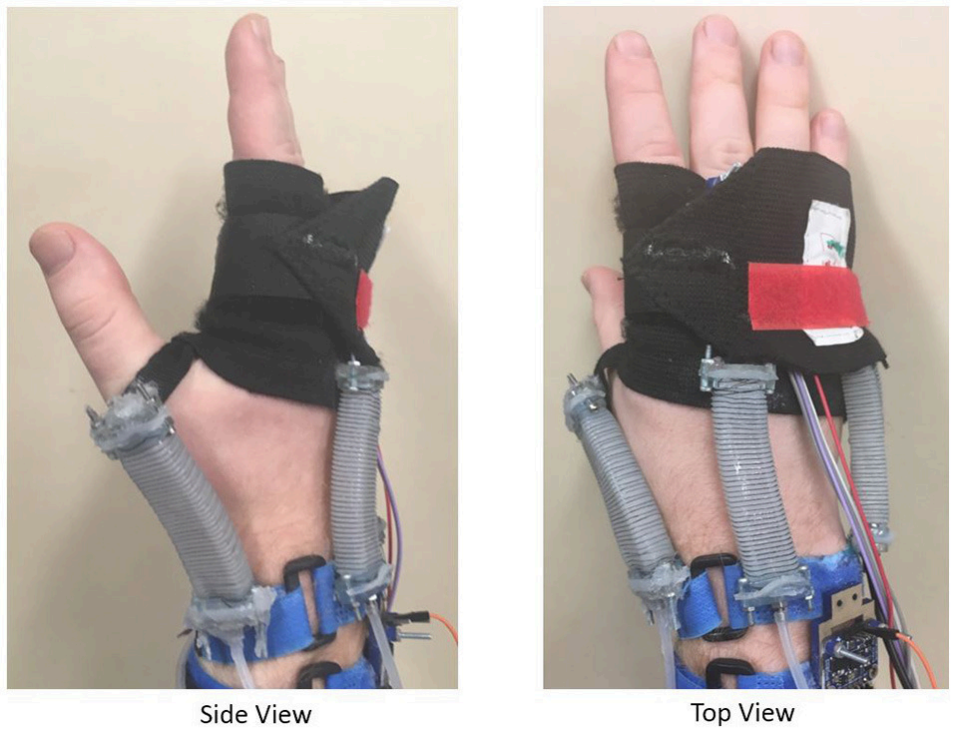
Views of the Haptic Wrist device worn by a user.

We created a device that mounts four rPAMs along the user's wrist, as shown in Figure 1. Under pressure, these actuators provide safe haptic torques on the wrist. To test the usability of this device, we created a simple path-following scenario that users underwent. In this scenario, users controlled a virtual agent using the angle of their wrist, with the goal of following a path as precisely as possible, with the haptic wrist device providing kinesthetic feedback. This device can be used as part of a soft haptic robotic arm teleoperation system, conveying forces to the user and also improving their performance via virtual fixturing, such as discussed by Rosenberg (1993).

A different type of soft actuator used in soft robotics the PneuNet. Presented by Ilievski et al. (2011), this actuator type consists of a series of connected chambers within a soft manifold. When pressurized, the chambers expand, causing the PneuNet to deform in a desired direction, usually out-of-plane bending. This bending can follow a similar path to the curling of fingers, and has been used in a soft pneumatic glove by Polygerinos et al. (2015). This system was intended for hand rehabilitation and only had a response time of around 2 s, not fast enough for real-time haptic feedback.

An example of a soft wrist device was discussed by Sasaki et al. (2005). This system uses pneumatic actuators similar to our rPAMs, but with an inextensible constraint layer that causes them to bend when pressurized. This work focused on EMG-enabled rehabilitation and only performed simple motions, with no precise control or angle feedback. In addition, the nature of the bending actuators used to apply forces meant that this system could only apply forces in a single plane, limiting its usability for haptic feedback. Similarly, a soft wrist device was created by Al-Fahaam et al. (2016) using both extensile and contractile McKibben muscles to actuate the user's wrist. Mounted on a glove [further analyzed in Al-Fahaam et al. (2018)], these actuators can apply high forces, although the authors did not investigate the control of the wrist.

Instead, the wrist device presented in this paper is both faster than other soft kinesthetic devices and safer, lower profile, and more flexible than existing rigid kinesthetic devices. We use precise pulse width modulation control of the soft actuators, which allows the device to provide real-time haptic feedback on the user's wrist.

Section 2 of this paper is devoted to discussing the fabrication of the haptic wrist device, including the actuators and the integrated sensors, as well as its physical capabilities. Section 3 focus on the setup and control required for the path-following trials we used for system verification. Section 4 shows the experimental results of the path-following trials, including both specific trajectories made by users and a aggregated analysis of Root Mean Square Errors (RMS Error). Finally, section 5 is the conclusion as well as a discussion of a possible use for this haptic wrist device as part of a larger system.

## 2. Fabrication

The wearable soft haptic wrist device described in this paper uses reverse pneumatic artificial muscles (rPAMs) to apply compliant forces to the wearers wrist. These rPAM actuators consist of a tube of silicone wrapped in a helix of thread. When pressurized, the thread provides a physical constraint that prevents the actuator from expanding into a sphere. Instead, the actuator simply extends, imparting compliant forces as discussed by Skorina et al. (2015) and Luo et al. (2015).

The compliant nature of these linear actuators makes applying compressive forces a difficult proposition. When doing so, the actuators have a tendency to buckle. To compensate for this, we have previously mounted these actuators antagonistically around a 3-D printed joint in a prestrained condition. Thus, when pressurized, the actuators relieve the pre-strain without buckling, allowing them to apply their antagonistic forces.

The rPAMs used in this paper were fabricated using a multi-step process. First, the hollow core of the actuator was created out of silicone rubber (DragonSkin 10) in a 3D printed mold. Next, nylon thread is wrapped in a uniform helical pattern around the actuator. The mold includes grooves around the outside, making it easy for the thread to be wrapped uniformly. To hold the thread in place, we applied a thin layer of uncured DragonSkin 10 over the outside.

To provide a seal for each end of the actuator, we used a technique we developed by Tao et al. (2015). This consists of a pair of acrylic plates sandwiching a flange at each end of the actuator, with the actuator fitting through the inner plate. These two plates were bolted together over the flange to form a tight seal. On one side of each actuator, a vent screw (a machine screw with a hole drilled through) was slotted though the outer plate, allowing pressure to be applied to the actuator chamber.

**Figure 2:**
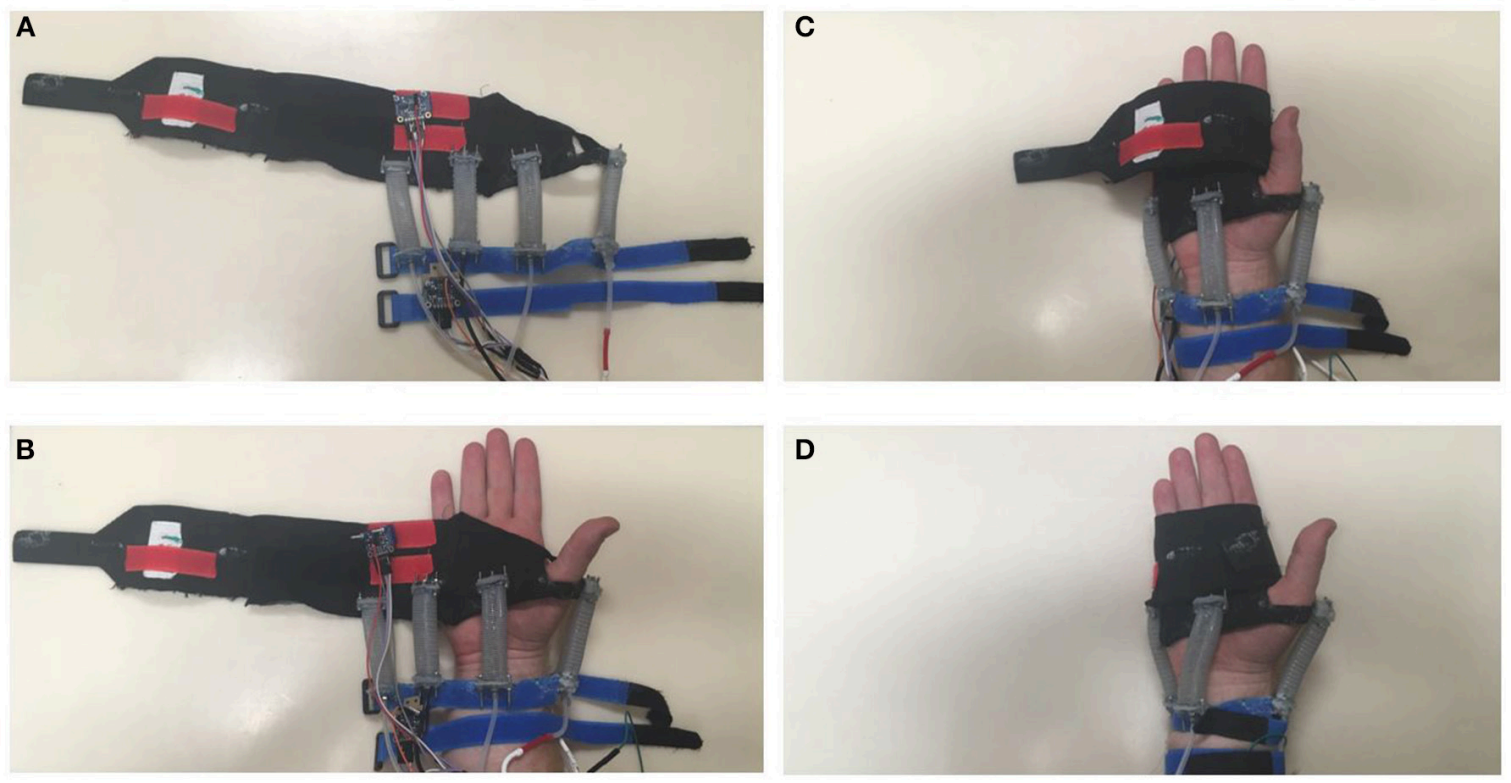
**(A)** The full Haptic Wrist Device, unattached to a user. **(B)** Step one in donning the device, put loop over the thumb. **(C)** Next step in donning the device, begin wrapping. **(D)** Fully wrapped, tighten the forearm straps.

Securing these actuators onto the forearm and hand was a challenge, especially considering the desired pre-strain in the rPAM actuators. We had to provide solid mounts for the actuators that would both resist actuator forces and stay put as the user moved their arm. After investigating several options, including nitrile gloves, velcro straps, and an elbow support, we settled on using different attachment methods at the hand and the wrist. To attach at the hand, we used a Flarico Hand Wrap, which is slotted over the thumb and than wrapped above the thumb and around the hand. The acrylic end-plates of the actuators were attached to the lowest layer of this wrap, so that they would be situated around all sides of the user's wrist when the device is worn. The material of the wrap is stretchy enough to adapt to different hand sizes. The donning process can be seen in Figure 2.

To mount the actuators on the forearm, we used a Bluecell Velcro strap secured below the user's wrist. Without tightening this Velcro strap to an uncomfortable level, this mount was still subject to sliding, but the bumps below the radius and ulna ensure that the actuators stay attached at a usable position. Again, the acrylic end plates of the actuators were glued to the strap, which allowed for a simple, effective mount.

In order for the system to measure the state of the user's wrist, we need to accurately measure the angle of the wrist in real time. To do this, we mounted a pair of inertial measurement units (IMUs) on the wrist device, one on the forearm and one on the back of the hand. We chose the BNO0055 9-axis absolute IMU for its small size, ease of use, and high reliability. This IMU performs all sensor fusion on-board, and only outputs its orientation which we use to calculate the angle of the wrist, as discussed later.

We mounted the IMU on the forearm by bolting it to an acrylic plate, and attaching the plate to Velcro strap. In order to ensure that this IMU remained stationary during motion, we added a second Velcro strap farther down the forearm, which was attached to the other side of the IMU attachment plate. The hand IMU was initially attached to the hand wrap using the same acrylic plate with the forearm IMU. However, initial user tests indicated that the fixed position of the IMU on the hand wrap was vulnerable to differences in user's hand size. To remedy this, we replaced the direct connection between the IMU plate and the hand wrap with a velcro connection. Thus, the IMU could be placed at a variety of points on the wrap to ensure its location coincided with the back of the user's hand. The IMU was placed on a lower level of the wrap, as shown in Figure 2, so the upper level could be used to hold the IMU in place. The entire device that is mounted on the user's wrist (excluding the circuitry and valves) has a mass of around 130 g.

### 2.1. Physical capabilities

**Figure 3:**
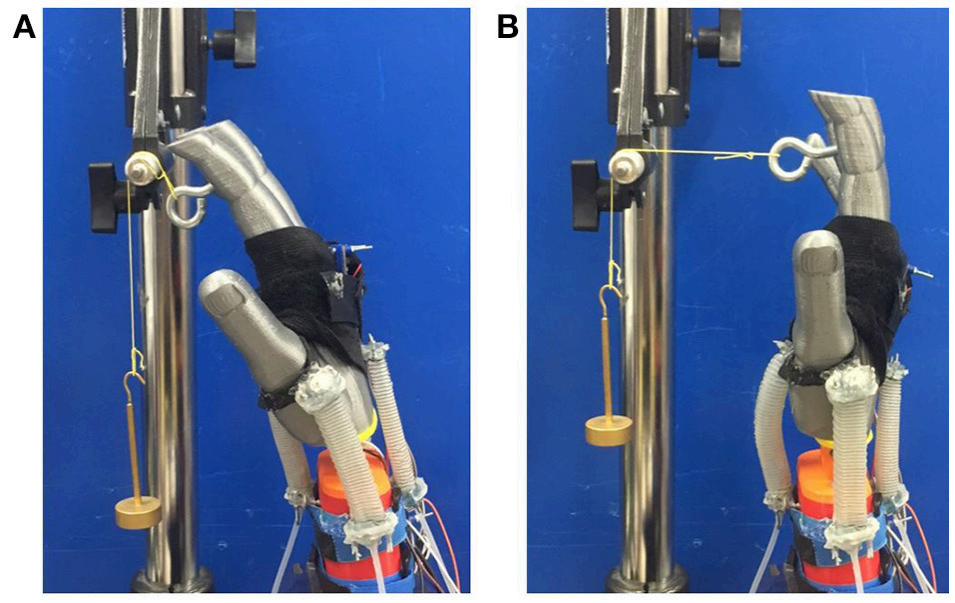
The Haptic Wrist force experimental. **(A)** torque applied, no pressure applied **(B)** torque applied, pressure applied to return the hand to the zero angle. Note, the pressurized actuator is against the palm, and is inflated and extended.

We created a test setup to verify the forces that the rPAMs can impart on the wrist and ensure that the torques are within a safe range. We 3-D printed a replica of a human hand and forearm. This model included a hinge joint at the wrist, which allowed it to pivot forwards and backwards. This joint also included a potentiometer for reliable and direct angle measurement. We applied known torques around the wrist joint, and adjusted the pressure in the corresponding actuator to return the joint to a neutral angle. This allowed us to determine the torque output of rPAM actuators on a user's wrist. The experimental setup can be seen in Figure 3.

We found that the maximum torque the actuators can apply while the hand is maintained at a straight pose is 0.14 Nm at 8 psi input pressure. If the actuators are given a higher pressure, they will start buckling, and the force applied to the wrist will not increase. This is significantly lower than the maximum wrist torque humans can sustain according to Morse et al. (2006) ensuring that our haptic device will be safe to operate regardless of any malfunctions. Thus, this was the pressure we used for all experiments.

## 3. Path following test

In order to test the usability of the haptic feedback provided by our device, we created a simple scenario where the user would use their wrist to control an agent operating in a simple virtual environment. We initially considered having the user control an agent in a maze or other obstacle-filled environment. However, we decided that the obstacles would add an element of problem-solving to any tests that were performed, adding noise to any direct analysis of the effect of the haptic feedback on user performance.

Instead, we chose to test the benefits of the haptic device on a simple path following task. This would allow the user to only focus on performing the intricate task as steadily as possible without having to worry about internal path planning. In addition, the nature of following an infinitely thin curve means that there is no performance ceiling, that there will always be room for the user to improve. Thus, there will always be room for a haptic device to assist the user.

**Figure 4:**
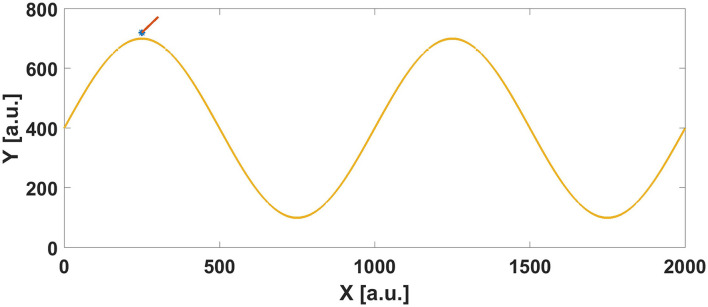
An example state the user would see when performing a path following experiment.

The environment consists of a path and an agent. We tested the usability of the system on both a straight line and a sinusoidal curve, both traveling left-to-right. The agent that the user controls within this environment is represented by a “^*^” icon. It has a forward velocity, represented by a line branching out from the agent position indicating the direction of travel. The length of this line was adjusted in conjunction with the velocity of the agent, to give some additional visual indication of velocity. A scene from the test environment is shown in Figure 4. The goal of the user was to follow the path as closely as possible while traveling all the way to the right side of the screen. We also instituted a time limit in the event that the user was traveling too slowly, to ensure that experiments were completed in a timely fashion.

### 3.1. Virtual agent control

At each simulation time step, the system calculates the motion angle and linear velocity of the agent from user input and iterate agent position on the screen. The user controls the agent using the angle of their wrist. In order to do this, it is first necessary to extract the wrist angle from the readings from the two IMUs. The control code running on the desktop computer receives global orientations of the IMUs in the form of quaternions. From there, we converted them to rotation matrices and used the following equation to find the rotation matrix of the wrist:

(1) $$\begin{array}{l} R_{D}=R_{2}R_{1}^{T}, \end{array}$$

where $R_{D}\in {ℜ}^{3\times 3}$ is the rotation matrix between the two IMUs, $R_{2}\in {ℜ}^{3\times 3}$ is the global rotation matrix to the wrist IMU, and $R_{1}\in {ℜ}^{3\times 3}$ is the global rotation matrix to the hand IMU. We convert this back to $D_{r}\in {ℜ}^{3\times 1}$, the raw local Euler angle state vector of the wrist. Because of the inconsistencies between human morphologies and the way the wrist device is wrapped around the hand, different users, or even the same user during different trials, would have different rest angles for their hand. Thus, we implemented a calibration routine every time the user puts on the device. This routine calculated the average *D*_*r*_ over 50 iterations of the user holding their hand loose and steady, this was used to calculate an offset value used during the subsequent experiments as follows:

(2) $$\begin{array}{l} D=D_{r}-D_{o}, \end{array}$$

where *D* ∈ ℜ^3 × 1^ is the adjusted angle of the wrist, *D*_*r*_ is the measured raw state discussed previously, and $D_{o}\in {ℜ}^{3\times 3}$ is the offset calculated during calibration. From this adjusted value of the state of the wrist, the algorithm calculates the angle of the next state using the following equation:

(3) $$\begin{array}{l} A_{i}=A_{i-1}-con\left(D\left(2\right)k_{a},-1,1\right), \end{array}$$

where *A*_*i*_ is the angle of the current state, *A*_*i*−1_ is the angle at the previous state, *k*_*a*_ is a sensitivity constant, *con*(.) is a constraint function, and *D* ∈ ℜ^3 × 1^ is the Euler angle state vector of the wrist in “yaw-pitch-roll” order. Thus, as this equation uses *D*(2), the angular velocity of the agent is controlled directly by the pitch of the user's wrist, that is the motion along the flexion/extension axis. The angular change per control loop is constrained between –1 and 1, while the angle itself is unconstrained. The control frequency is around 15 Hz, which means that the maximum rotational velocity is around 2.5 revolutions per second. For all experiments performed, *k*_*a*_ = 1.

The human wrist has three degrees-of-freedom (DoF), and we use one of them to control the angular velocity of the agent. Thus, we decided to study the use of an additional DoF in the wrist to control the linear velocity of the agent. The user could speed up and slow down the agent, depending on how well they were following the line. This would allow an additional metric of velocity to be examined in order to gauge user performance. The velocity of the agent was controlled by:

(4) $$\begin{array}{l} V_{i}=con\left(V_{i-1}+con\left(D\left(1\right)k_{v},-0.3,0.3\right),15,2\right), \end{array}$$

where *V*_*i*_ is the linear velocity at the current state, *V*_*i*−1_ is the linear velocity at the previous state, and *k*_*v*_ is a sensitivity constant. As this equation uses *D*(1), the linear acceleration is controlled directly by the yaw of the wrist, that is, the lateral motion in the radial/ulnar direction. We also observed that users could more easily move their hands in the ulnar direction (toward the little finger) than in the radial direction (toward the thumb). Thus, we used a different value for *k*_*v*_ depending on which direction the user was turning their wrist, as follows:

(5) $$k_{a}=\{\begin{array}{ll} 15, & \text{if }D(1)<-0.01\\ 7, & \text{if }D(1)>0.08\\ 0 & \text{otherwise}. \end{array}$$

This equation also creates a deadzone, where it would theoretically be easier for the user to steer the agent without accidentally changing its velocity. However, initial experiments indicated that this method of controlling the agent velocity was still difficult for most users. Some users found that the mental coupling between the two axes of rotation in the wrist made it difficult to control one without changing the other. Thus, we looked into different ways of controlling the linear velocity of the agent using the user's body. We settled on controlling the linear velocity of the agent directly using the angle of their forearm. This would decouple the two DoF, while using our already existing hardware. The velocity control equation for this method is written as:

(6) $$\begin{array}{l} V_{i}=con\left(E_{2}\left(1\right)k_{v2},20,3\right), \end{array}$$

where $E_{2}\in {ℜ}^{3\times 1}$ is the global Euler angle of IMU2 (the IMU mounted on the forearm), and *k*_*v*2_ is a sensitivity constant. Thus, if the user held their hand and forearm with the little finger downwards, they could directly control the velocity of the agent by moving their forearm up and down. For all of these experiments, we used *k*_*v*2_ = 30. The velocity was constrained between 20 and 3. This comes out to a maximum velocity of one sixth of the horizontal length of the environment per second, and the minimum velocity being one fortieth of the horizontal length of the environment per second. Thus, the user would reach their maximum velocity when the arm was held at around 40° above horizontal

### 3.2. Feedback control

In order for the system to guide the user to the desired path, we created an algorithm to determine the pressure in each rPAM. To do this, we calculated how far the agent was from the nearest point on the path. For faster computation, this distance was only calculated for the 400 nearest points on the horizontal axis. The distance to each of these points was calculated, and the shortest distance was determined. Once that point was determined, we calculated the angle needed for the agent to reach that point, as well as the angle of the path at that point. The desired angle for the agent was then calculated as a weighted average between these two angles:

(7) $$\begin{array}{l} A_{d}=\alpha a_{1}+\left(1-\alpha \right)a_{2}, \end{array}$$

where *A*_*d*_ is the desired angle, *a*_1_ is the angle for the agent to reach the nearest point on the path, and *a*_2_ angle of the path at that point, and α ∈ [0, 1] is the weight. α becomes smaller as the minimum distance to the nearest point on the path becomes smaller, and is calculated using the following equation:

(8) $$\begin{array}{l} \alpha =\min\left(\frac{m}{200},1\right) \end{array}$$

where *m* is the minimum distance to the desired path. Thus, if *m* > 200, α = 1 and the desired angle is equal to the angle between the agent and the nearest point on the path. As the virtual agent approaches the path, the desired angle becomes more and more aligned with the direction of the path. Entirely following the desired angle, an agent would asymptotically approach the desired path.

Using the desired angle, we calculate the angle error *A*_*e*_ using the following equation:

(9) $$A_{e}=\{\begin{array}{ll} A_{d}-A_{i}, & \text{if }|A_{d}-A_{i}|\text{ ​}>dz\\ 0 & \text{otherwise}. \end{array}$$

where *dz* is the deadzone. Thus, when the user is pointing their wrist within a certain threshold of the desired angle, it will be considered to be perfectly accurate. From here, the control command to the valves is calculated as:

(10) $$\begin{array}{l} u_{1}=con\left(50+k_{au}A_{e},0,100\right) \end{array}$$

where *u*_1_ is the angle control input,*k*_*au*_ is the sensitivity constant, and *A*_*e*_ is the angle error. We used *k*_*au*_= 30 for all experiments. This equation generates a control input between 0 and 100 with 0 resulting in full actuation in one direction and 100 resulting in full actuation in the opposite direction.

We also wanted to investigate the ability of the haptic feedback to help the user regulate their velocity. When the user is following the desired path with a high level of accuracy, we would want them to speed up to reach the end of the path faster. On the other hand, if the user has a large error, we would want them to slow down to better focus on returning to the path. To this end, we implemented the following feedback control law for velocity:

(11) $$\begin{array}{l} V_{d}=\max\left(18-|A_{e}|k_{vu},3\right), \end{array}$$

where *V*_*d*_ is the desired velocity, and *k*_*vu*_ is the sensitivity constant. For all experiments performed, *k*_*vd*_ = 12. Through this equation, the desired velocity ranges between 18 and 3, and is linearly related to the angle error. When the user is pointing in the desired direction, the desired velocity will be high, telling the user to speed up to return to the path or speed along it toward the goal. However, when the user is not pointing in the correct direction, the desired velocity will be low, telling the user to slow down. We calculated the velocity error in a similar manner to equation 9, with a corresponding deadzone of 3, and calculated the command to the controller by

(12) $$\begin{array}{l} u_{2}=con\left(50+k_{vu}V_{e},0,100\right), \end{array}$$

where *u*_2_ is the velocity control input, *V*_*e*_ is the error between the desired velocity and the actual velocity, and *k*_*vu*_ is a sensitivity constant.

This feedback on the lateral motion of the hand was still used when the velocity was being controlled by the angle of the forearm. As the hand was directed to be held sideways, the changes in the angle of the arm would be in the same plane as the lateral motion of the hand. Thus, the lateral haptic cues on the hand would still indicate to the user the correct direction to move their arm, even if the forces were not directly affecting the joint used for control.

#### 3.2.1. Valve control

An Arduino Mega control board receives commands from Matlab and directly controls 30 Hz pulse-width-modulation (PWM) signals of the four digital valves connected to the rPAM actuators. Several different control schemes were evaluated, including our previous method of both valves operating antagonistically, as in Luo et al. (2017). The final control scheme we settled on is as follows:

For *u*_*i*_>50:

(13) $$\begin{array}{l} \begin{array}{c} c_{1}=0,\\ c_{2}=2\left(u-50+c_{O}\right)\times 255/100, \end{array} \end{array}$$

for *u*_*i*_ < 50:

(14) $$\begin{array}{l} \begin{array}{c} c_{1}=2\left(50-u+c_{O}\right)\times 255/100,\\ c_{2}=0, \end{array} \end{array}$$

and if *u*_*i*_ = 50, then:

(15) $$\begin{array}{l} \begin{array}{c} c_{1}=0,\\ c_{2}=0, \end{array} \end{array}$$

where *c*_1_ and *c*_2_ are 8-bit duty cycles sent directly to the two valves and *c*_*O*_ is a command offset. *c*_*O*_ is included because at low duty cycles (*c* < 30) the commands will be too fast to register, and the valve will not actuate. Thus, *c*_*O*_ was set to 15 for all experiments. This saturation behavior also occurs where *c*>70, but precision is not necessary when the error is high. These equations were used for pairs of actuators on each actuated wrist axis.

## 4. Experimental results

We performed a range of path-following experiments with users^1^. In order to collect data under a wide range of circumstances, we studied all combinations of the type of path, the type of feedback used, and the variability of velocity, for a total of ten experiments (velocity feedback was not used when the velocity of the agent was fixed). When a fixed velocity was used, we set it at 50 a.u. per second, or about one twenty-fourth of the horizontal length of the environment. The two paths used were a straight horizontal line and a sine wave with amplitude 300 a.u. and a period of 1,000 a.u. The sine wave trajectory is shown in Figure 4, with two periods taking up the entire length of the environment. Pressure for the haptic feedback was provided though the building and regulated down to 8 psi. Initial positions were on the desired position at *x* = 250 but with an initial angle $\frac{\pi }{2}$, forcing the users to immediately correct their trajectory to match the path. This was particularly relevant on the horizontal line following experiments, where if the initial angle was along the direction of the path, the user wouldn't have to do anything in order to follow it successfully. An example experiment, with haptic feedback and fixed velocity following a sine path, can be seen “Video 1” of the Supplementary Materials.

We first helped the users don the wrist device. We tightened the wrist device, ensuring it was tight enough to keep the actuator mounts from shifting while receiving verbal confirmation that it was not uncomfortably tight.We gave the users instructions about the testing scenarios and about how to control the virtual agent, then asked new users to practice control of the agent without feedback. After this, users were given 10 experiments in random order, with a brief pause in between each where the properties of the next experiment were listed, allowing the users to prepare themselves. Before each set of experiments (or practice runs), the users were asked to hold their wrist at a comfortable neutral pose. This was used as the zero angle by the simulation, compensating for differences in orientation of the IMUs when mounted on each user.

We performed this set of 10 different experiments with nine different volunteers (mostly males ages 21–28) from our research group, with some users performing the random set multiple times. We did an initial run of 18 experiments. In this first set of experiments, we used the lateral motion of the wrist to control the velocity when velocity was variable (see Equation 4) and a deadzone *dz* = 4.5^*o*^ (0.078 rad). We measured the root-mean-square (RMS) error for the user for each of these trials, as well as their average horizontal velocity. The RMS errors (RMSE) were calculated using the distance to the nearest point calculated during the control, while the average velocities were calculated using the final x-coordinate and the final time recorded for a given trial. The means and standard deviations for the RMS errors for each experiential type are shown in Table 1.

**Table 1:** The errors for all the path following experiments performed with deadzone *dz* = 9^*o*^ (0.156 rad).

**Linear Path**			**Sine Path**	
	**Const. Velocity**	**Var. Velocity**	**Const. Velocity**	**Var. Velocity**
No Feedback	17.3 ± 14.0	43.5 ± 39.5	**40.1** ± **18.2**	88.2 ± 59.0
Angle Feedback	15.2 ± 8.5	24.4 ± 16.1	**31.4** ± **14.9**	76.6 ± 52.4
Angle & Velocity Feedback	–	32.9 ± 25.3	–	101.5 ± 58.6

*Parameters investigated were the quantity of haptic feedback (no feedback, feedback on the agent angle, and combined feedback on both the agent angle and velocity), the velocity of the agent (fixed vs. controllable), and the path type (straight vs. sinusoidal). When variable velocity was used, it was controlled using the Radial/Ulnar deviation of the wrist. Errors are presented in the form Mean ± Standard Deviation and in arbitrary units [a.u.]. The sine path/constant velocity data is bolded to highlight the statistically significant improvements brought on by the haptic angle feedback*.

The first observation we can make from this data is that the addition of feedback provides a noticeable improvement for the sine wave experiments, with and without a variable velocity. In particular, there is around a 30% improvement in the RMSE of the fixed-velocity sine wave following, as well as a smaller improvement during variable velocity experiments. Performing a *T*-Test, we can see that this improvement is statistically significant for the fixed-velocity trial (*p* < 0.05).

**Figure 5:**
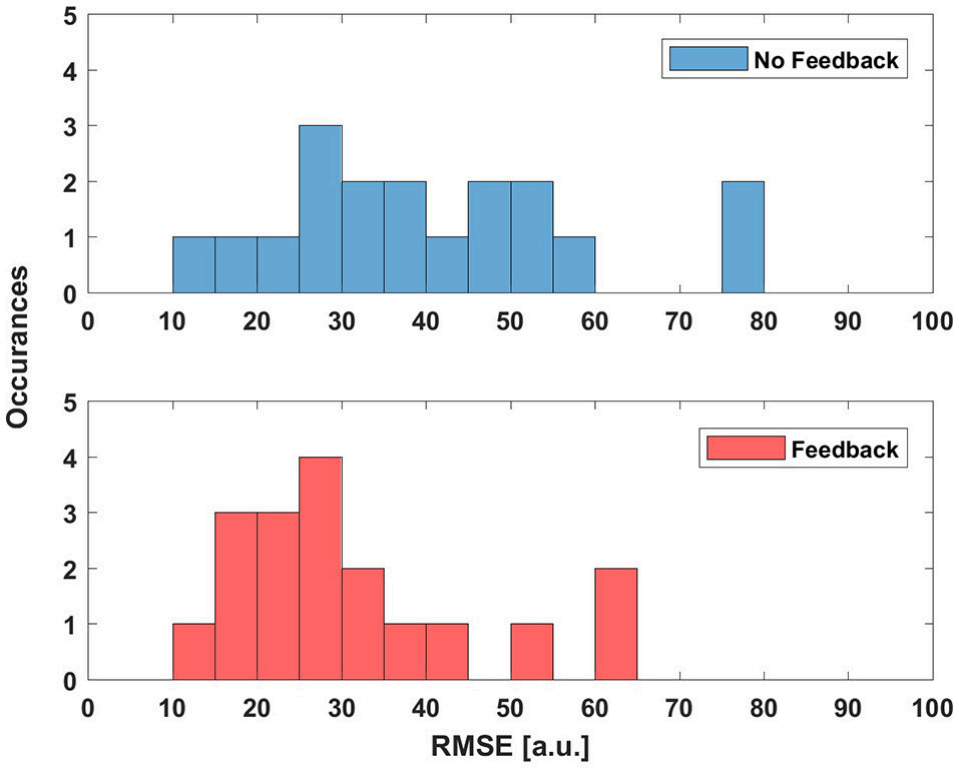
A histogram of user RMS Error for users following a sinusoidal path with and without haptic feedback. The agent velocity was fixed and the deadzone *dz* = 4.5^*o*^ (0.078 rad).

A histogram comparison of the fixed-velocity trials can be seen in Figure 5. The data without haptic feedback was fairly uniform, with some users struggling to control the agent. Haptic feedback helped users follow the path more accurately, though the wrist device was not effective enough to help users reduce their RMS error to below 10^*o*^.

**Figure 6:**
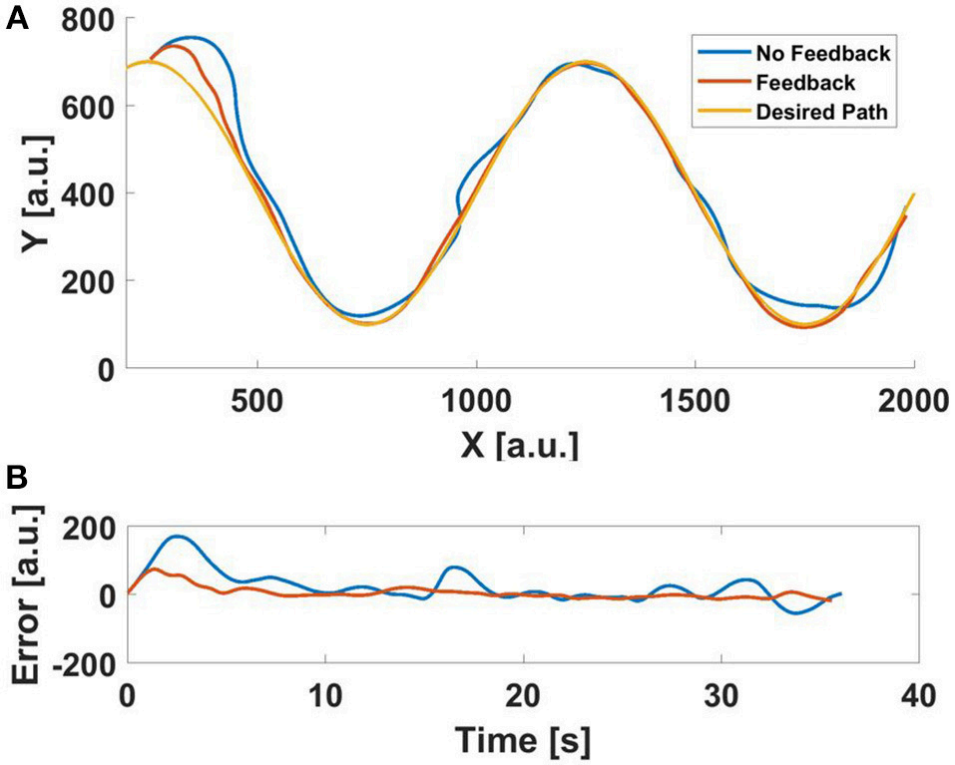
An example of a user's performance with and without haptic feedback when following a sine wave path with a deadzone *dz* = 4.5^*o*^ (0.078 rad). **(A)** the trajectory **(B)** error.

One example of a trajectory pair, comparing a single user's performance with and without feedback in a sine-following fixed-velocity scenario, can be seen in Figure 6, where we show both the full trajectories and the error. We can see that the feedback pulls the user away from their initial divergent trajectory much faster. The changing nature of the sine path fosters error on the user, error that the haptic device is able to help correct. In addition to providing correcting forces, some users also reported that the perceived vibration (resulting from the PWM pressure control of the valves) kept them focused on the task when the agent was starting to diverge.

**Figure 7:**
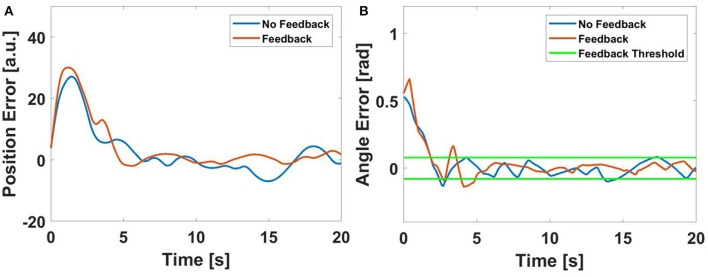
An example of a user's performance with and without haptic feedback when following a linear path with a deadzone *dz* = 4.5^*o*^ (0.078 rad). **(A)** the error with respect to the desired position for each path **(B)** The error with respect to desired angle chosen by the haptic algorithm (as calculated by Equation 7).

In contrast, an example comparison of a single user's performance following a straight path can be seen in Figure 7. This figure shows both the error (which, as the desired path is straight, represents the trajectory), but also the error with respect to the desired angle (see Equation 7). In this case, we can see that the feedback does not help. The feedback seems to drive the user into over-correcting. In addition, because the linear trajectory is easier to follow, much more of the motion occurs within the deadzone. Thus, feedback plays a much smaller role in affecting performance, which is supported by the aggregate data for the straight line following experiment.

We saw that the haptic feedback provided a slight improvement for the variable-velocity sine following experiments, but the variance in the data was too large to show any statistical significance. This applied to other experiments as well, and was not affected by the haptic velocity control. Users reported that using both DoF on the wrist was difficult, such as increasing the velocity without turning, and vice-versa. This is despite initial calibration experiments that showed that the two degrees of freedom were not coupled. Within the cognitive burden of the path following task, users found it hard to perform pure motions that are decoupled in the 2 DoF we measure through our soft haptic wrist device.

To circumvent this problem, we investigated an alternate method of controlling the linear velocity of the agent. As discussed previously, we performed experiments using the angle of the forearm to directly control velocity (see Equation 6). In addition, we also shrunk to *dz* = 3^*o*^ (0.052 rad), with the goal of improving feedback-performance on the straight line trials. We performed 11 additional experiments under these conditions, with all other aspects of the experimental processes remaining the same. The results of these experiments can be seen in Table 2.

**Table 2:** The errors for all the path following experiments performed with deadzone *dz* = 3^*o*^ (0.052 rad).

**Linear Path**			**Sine Path**	
	**Const. Velocity**	**Var. Velocity**	**Const. Velocity**	**Var. Velocity**
No Feedback	18.6 ± 13.7	24.5 ± 13.2	**36.2** ± **13.3**	100.4 ± 121.0
Angle Feedback	12.5 ± 4.0	18.4 ± 12.1	**26.1** ± **12.4**	50.2 ± 20.2
Angle & Velocity Feedback	–	16.7 ± 6.2	–	47.1 ± 13.5

*Parameters investigated were the quantity of haptic feedback (no feedback, feedback on the agent angle, and combined feedback on both the agent angle and velocity), the velocity of the agent (fixed vs. controllable), and the path type (straight vs. sinusoidal). When variable velocity was used, it was controlled using the angle of the forearm. Errors are presented in the form Mean ± Standard Deviation and are in arbitrary units [a.u.]. The sine path/constant velocity data is bolded to highlight the statistically significant improvements brought on by the haptic angle feedback*.

**Figure 8:**
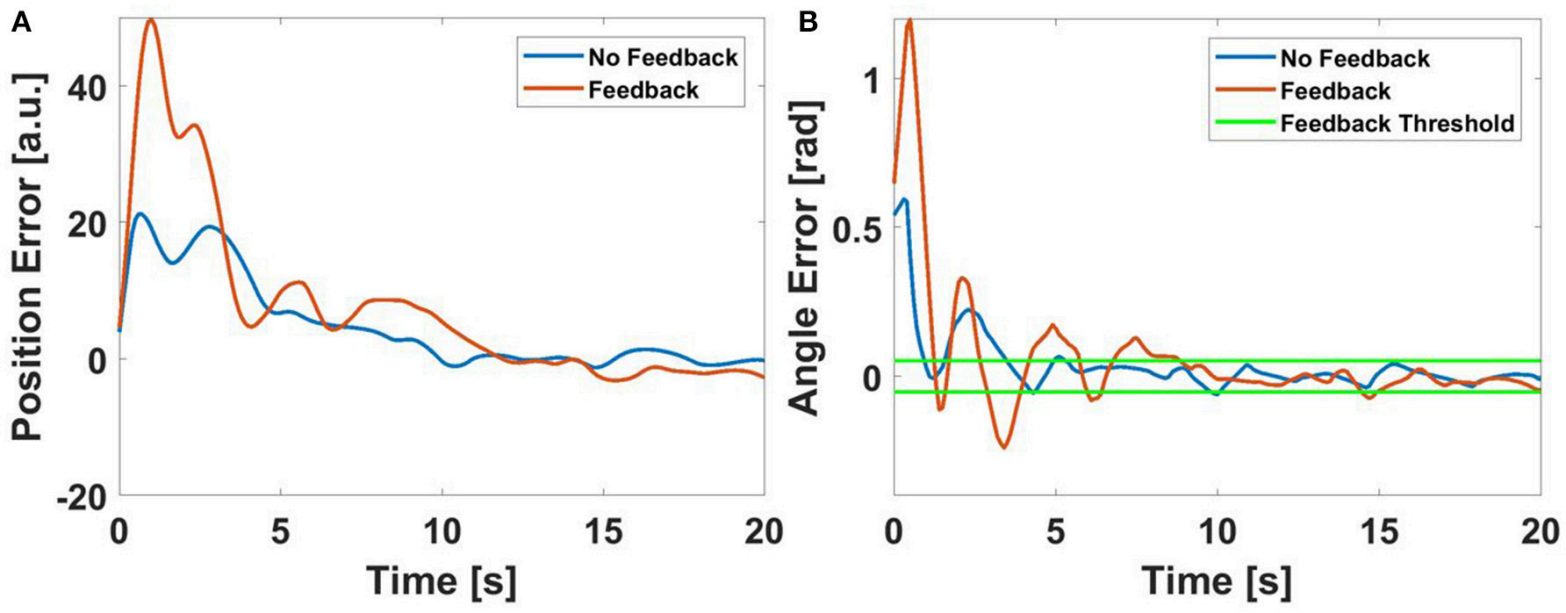
An example of a user's performance with and without haptic feedback when following a linear path with a deadzone *dz* = 3^*o*^ (0.052 rad). **(A)** the error with respect to the desired position for each path **(B)** The error with respect to desired angle chosen by the haptic algorithm (as calculated by Equation 7).

From this table, we can see that the changes did improve the effectiveness of the haptic feedback, but not in a statistically significant manner. For the fixed velocity horizontal line following, the decrease in the size of the deadzone did not seem to improve the effects of the haptic feedback. Even when the rPAM in the haptic glove are applying a very small amount of force, the vibration resulting from the PWM pressure control may be causing the users to overcorrect. An example plot of the angle error can be seen in Figure 8. While the addition of haptic feedback speeds up the user's ability to approach the desired path, it seems to increase the oscillations once the user is near the target trajectory. The haptic feedback seems to cause the user to overcorrect, zig-zagging around the deadzone.

We also found that the alternate method of controlling velocity was ineffective at increasing velocity-control performance. Users reported that the cognitive burden of controlling both degrees of freedom with their wrist was replaced with the burden of focusing on two entirely separate muscle groups simultaneously: wrist and elbow. In addition, users would often forget about the importance of arm position during fixed-velocity trials, resulting in a high initial velocity and then greater errors during a variable-velocity trial that followed.

However, this new round of data continues to show the improvements the haptic device can bring to fixed-velocity, sine-wave trajectories at *p* < 0.05. Combined with the initial data set, we can conclude that over all experiments the haptic feedback provided an improvement of around 30% with a *p* < 0.01. Our haptic wrist device is useful for providing larger-level feedback, for users following variable dynamic trajectories, while it struggles to provide effective feedback for more precise tasks.

## 5. Conclusion

Haptics can provide new avenues for human users to communicate with computers and robots. In particular, soft wearable haptics can conform to the user's body and apply feedback forces and torques while still remaining flexible to user motion and easily adapting to variations in user body dimensions. We created a soft haptic wrist device constructed using reverse pneumatic artificial muscles (rPAMs). This device is capable of sensing the wrist state and applying 2 degrees-of-freedom haptic cues with torques no higher than 0.15 Nm.

We created a scenario where the user moved their wrist and arm to control an agent following a path in a virtual environment. The user was capable of controlling the angular acceleration of the agent via the position of their wrist along the sagittal plane. The forward velocity of the agent was either fixed, controlled by the transverse motion of the wrist, or controlled directly by the angle of the forearm. The haptic wrist feedback device provided gentle torques, directing the user toward the desired path while not overpowering them.

We performed a number of experiments under various conditions, and found that the haptic feedback device was a significant benefit in helping the user follow non-linear paths with a fixed velocity, making it the first soft robotic device capable of performing real-time kinsethetic feedback. In addition, the device as a whole is safer, lighter, more form-fitting, and adaptable to different users than an equivalent rigid device would be.

However, when following linear paths, the haptic feedback was not precise enough to provide any significant performance improvements, often causing users to overcorrect. Under variable velocity conditions, haptic feedback provided small but statistically insignificant performance improvements. Velocity was difficult to control under both control schemes test. Velocity represented another property for users to think about, diverting their attention from steering, a particular concern for the forearm velocity control where users had to use different parts of their body to control the agent. This was less of a factor when using the different bending directions of the wrist, but for that scheme users struggled un-coupling the two wrist axes. An increase and decrease in velocity would often cause users to veer off the path. Skilled users were able to effectively utilize these multiple degrees-of-freedom simultaneously, but less skilled ones were not. This, combined with difficulty of path-following at higher speeds, resulted in a much higher variance in the data and eliminated any statistical significance.

One of the main weaknesses of the proposed device is minimum force that the actuators can apply. The nature of the PWM pressure control allows for fast response times, but valves have trouble operating consistently at very low or very high duty cycles. This meant that the actuators struggle to provide useful kinesthetic feedback at low errors (as demonstrated by the only marginal improvements under horizontal line-following conditions). This could be mitigated through a more complex pressure-application scheme, such as through pistons, though any system with the same response time and more precision as our will likely be significantly bulkier and more expensive.

One interesting aspect of our experiments was the discrepancy between the versatility of the wearable haptic device and the user's ability to control the virtual agent. The haptic device was able to measure the local wrist angle regardless of the global orientation, which would theoretically allow the user to control the agent regardless of the orientation of their hand. However, we observed that users struggled to control the agent effectively except in certain preferred orientations, where the motion of the wrist matched the motion of the agent by an intuitive mapping.

This leads to some of the ongoing aspects of this project. We would like to use the haptic wrist device as part of a more complicated wearable haptic system. We plan on integrating this device into a haptic system used to teleoperate a robotic arm for 3-D manipulation tasks. In future work, the device introduced in this paper will be used to simulate gravity and contact forces on the user's wrist, as well as provide haptic cues to improve teleoperation performance. In order to fully apply haptic forces to a user's arm, techniques would have to be developed to apply forces to the user's elbow and shoulder, something the actuators in this paper could not extend and contract enough to effectively accommodate. In addition, this work could be used as part of a self-contained wearable device. It would function as a low-profile force-feedback joystick, allowing a worker to control machinery while in the field. This would require a personal pressure source. Though pumps outputting the pressures used this work are available that can be incorporated into a wearable device, the noise they generate is a problem that would have to be overcome.

## Author contributions

CO came up with the idea for the rPAM actuators and their use for haptic feedback. ML developed the techniques for the actuator fabrication, and created the valve control circuitry. ES developed the haptic control algorithms, fabricated the device, oversaw user experiments, and analyzed the data. ES, ML, and CO wrote the paper.

### Conflict of interest statement

The authors declare that the research was conducted in the absence of any commercial or financial relationships that could be construed as a potential conflict of interest.

## Footnotes

^1^This study was carried out in accordance with the recommendations of Federal Regulations 45 (CFR46) with written informed consent from all subjects. All subjects gave written informed consent in accordance with the Declaration of Helsinki. The protocol was approved by the WPI Institutional Review Committee.
